# Measuring Vestibular Contributions to Age-Related Balance Impairment: A Review

**DOI:** 10.3389/fneur.2021.635305

**Published:** 2021-02-09

**Authors:** Andrew R. Wagner, Olaoluwa Akinsola, Ajit M. W. Chaudhari, Kimberly E. Bigelow, Daniel M. Merfeld

**Affiliations:** ^1^School of Health and Rehabilitation Science, The Ohio State University, Columbus, OH, United States; ^2^Department of Otolaryngology—Head and Neck Surgery, The Ohio State University, Columbus, OH, United States; ^3^Department of Mechanical and Aerospace Engineering, The Ohio State University, Columbus, OH, United States; ^4^Department of Mechanical and Aerospace Engineering, University of Dayton, Dayton, OH, United States; ^5^Department of Biomedical Engineering, The Ohio State University, Columbus, OH, United States

**Keywords:** vestibular, balance, aging, perceptual threshold, falls

## Abstract

Aging is associated with progressive declines in both the vestibular and human balance systems. While vestibular lesions certainly contribute to imbalance, the specific contributions of age-related vestibular declines to age-related balance impairment is poorly understood. This gap in knowledge results from the absence of a standardized method for measuring age-related changes to the vestibular balance pathways. The purpose of this manuscript is to provide an overview of the existing body of literature as it pertains to the methods currently used to infer vestibular contributions to age-related imbalance.

## Introduction

In the United States, 28–49% of older adults (≥65 years old) fall each year (1, 2). Falls are the most common cause of accidental death in older adults (1), and in non-fatal cases, the costs to manage fall-related sequelae are poised to exceed 55 billion dollars in 2020 (3, 4). Despite a great deal of research in the area of fall prevention (5, 6), falls, and fall-related deaths continue to rise (1) and the growing age of the United States population (4) is likely to perpetuate this trend. Although a fall can result from a variety of environmental or physiological causes (7–9), a principal contributor to falls among older adults is an age-related decline in balance (8–12). In this paper, we define the “human balance system” as the sensorimotor system that permits us to stand upright (and walk/run) even though the passive biomechanics are unstable, being comprised of interconnected inverted pendulums that are each unstable. Balance reflects “…the ability to maintain equilibrium in a gravitational field by keeping or returning the center of body mass over its base of support” (13) and is the critical component for successful (a) quiet stance, (b) compensatory postural reactions, and (c) anticipatory postural responses. It follows, then, that imbalance (i.e., the lack of balance) be defined as the inability to maintain equilibrium during quiet stance and to anticipate or respond appropriately to postural perturbations. Imbalance can be influenced by a myriad of age-associated declines in sensorimotor function, including somatosensation (14–16), vestibular function (17), vision (18), cognition (19), and strength (20, 21). Therefore, balance tests are often used to indirectly quantify these different sensorimotor contributors with different balance tests quantifying different balance elements. To develop targeted, personalized interventions to combat this epidemic of falls, it is crucial that we develop methods that isolate the primary contributors to an older adult's imbalance and fall risk.

Although deficits in any one of the aforementioned physiologic systems could likely contribute to imbalance, this review aims only to summarize studies that attempt to quantify contributions of the vestibular system to age-related balance impairment. The rationale for this decision is as follows: (1) Recent estimates suggest that falls due to vestibular impairment represent between the 3rd and the 10th leading cause of death amongst older adults (22), (2) 35% of adults over the age of 40 show evidence of vestibular dysfunction (23) and amongst adults that report unsteadiness, one third also report symptoms of dizziness (24), (3) increased noise within the vestibular system (assayed by roll tilt perceptual thresholds) has been shown to mediate 46% of age-related balance impairment in asymptomatic adults above age 40 (17), and (4) vestibular function has been shown to be modifiable with rehabilitation (25–32). Thus, we embark on this effort because despite the overabundance of evidence supporting both age-related declines in vestibular function (22, 23, 33–36) and a significant contribution of the vestibular system to postural control (37, 38), an understanding of the specific contributions of the vestibular system to age-related balance declines is lacking; recent proceedings from a National Institutes of Health workshop identified this as a critical gap in our understanding of fall prevention (39).

In an effort to address this critical knowledge gap, this manuscript aims to review the methods currently used to quantify vestibular contributions to age-related balance dysfunction including (I) measures of the peripheral vestibular end organs (i.e., otoliths and semicircular canals) that correlate with balance, (II) external stimulation of the descending vestibular pathways, (III) objective balance assessments, and (IV) measures of perceived vertical. We also highlight a recent line of research relating vestibular perceptual thresholds to age-related balance declines (V). We note that several areas of vestibular function (e.g., spatial cognition, navigation, and autonomic function) likely relevant to falls, but not directly relevant to balance, were intentionally omitted from this review given our focus on vestibular contributions to age-related imbalance. An indirect goal of this review is to motivate researchers and clinicians to develop novel methods for quantifying specific contributions of the aging vestibular system to balance impairment.

### Tests of the Peripheral Vestibular Labyrinth

#### Semicircular Canals

Caloric irrigation is a gold standard for diagnosing a unilateral impairment in the horizontal semicircular canal and superior vestibular nerve (40), yet few studies have investigated its ability to predict balance impairment in healthy (i.e., without vestibular pathology) older adults. Jacobson et al. (41) compared the results of caloric irrigation to performance on a Modified Romberg Balance Test (MRBT) in a group of 45 asymptomatic adults over the age of 40—the age at which balance and vestibular function are thought to begin to decline (22, 23). Of the 20 subjects found to have an impaired caloric response, only 11 showed evidence of imbalance and of the 25 subjects found to have a normal caloric response, nine subjects demonstrated concurrent balance impairment (41). Similarly, in a sample of adults with balance impairment, Whitney et al. (42) identified an abnormal caloric response in only 13% (4/13) of recurrent fallers, while 44% (23/52) of subjects without a positive fall history demonstrated an abnormal caloric response. Collectively these results suggest that impairments in the low frequency vestibulo-ocular reflex (VOR) may not reflect impairment in the descending balance pathways.

Rotational chair testing measures bilateral horizontal canal function by quantifying the phase and gain of the mid to high frequency yaw VOR (43). Peterka and Black (35) found that although balance clearly declined with age, the incidence of abnormal VOR gain (±3 SD from the mean), was similar between adults with and without evidence of balance dysfunction (36). In a sample of healthy older adults, Baloh et al. (34) showed an age dependent decline in the VOR over a 5-year period despite stable balance performance. When these same participants were reassessed after an 8–10-year period, a multivariate regression model showed that although the VOR continued to decline with age, MRI evidence of white matter hyperintensities, visual acuity, and auditory function were the only variables significantly associated with changes on the Tinetti Gait and Balance (TGB) scale (a clinical measure of balance performance) (44). A subsequent study by Kerber et al. (45) did however identify a significant correlation between changes in the gain and phase of the low-frequency (0.05 Hz) VOR and TGB scores after a 9-year period. While this correlation remained after correcting for age (*p* < 0.001), the authors suggest that this relationship likely represents a common age-related central pathology, potentially white matter hyperintensities, that affect both balance and the VOR, rather than a causal relationship between age-related VOR declines and balance impairment (44–46). The aphysiologic vestibular stimulus (i.e., lacking head motion) during caloric irrigation (41) and the restricted frequency range of the vestibular stimuli during both caloric irrigation (0.006 Hz) and rotational chair testing [0.01–1.0 Hz (43)], relative to typical human motion [~0.8–3.2 Hz (47, 48)], pose possible explanations for the limited association between these standard assessments of canal function and balance impairment among older adults.

The video head impulse test (vHIT) instead measures canal function through the delivery of passive head rotations (i.e., impulses) that reach accelerations [~3,000°/s/s (49)] and frequencies [~6 Hz (50)] consistent with naturalistic human motion (48, 51). Over the past decade vHIT has been used to lateralize peripheral vestibular lesions (52–54) and to differentiate peripheral from central causes of an acute vestibular syndrome (55–57). However, the use of vHIT in healthy (i.e., without occult vestibular pathology) older adults has received little attention by comparison. Anson et al. (15) showed that a yaw vHIT VOR gain of <0.80 bilaterally (eye velocity <80% of head velocity) was associated with greater amounts of postural sway in the eyes closed, compliant standing (i.e., foam) condition of the MRBT. Anson and colleagues subsequently showed that for every 0.1 decrease in VOR gain, the likelihood of failing (i.e., falling prior to 40 s) this same test increased by 8% (58). Compared to the low frequency range of calorics and rotational chair testing, the high frequency content (1–6 Hz) of impulsive testing provides a stimulus to the vestibular system that is more representative of the demands of typical human locomotion [0.8–3.2 Hz (51)], providing a potential explanation for its superior association with balance impairments among older adults (50). Yet, in a group of 183 healthy older adults, Xie et al. (59) showed that yaw vHIT VOR gain was not associated with one's gait speed, narrow base of support balance, or the ability to rise from a chair. Thus, high frequency horizontal canal function, assayed using vHIT, may be most relevant for maintaining quiet stance in situations where visual and proprioceptive feedback are made less reliable (e.g., standing in a hallway at night on a thick rug while feeling for a light switch).

Whereas vHIT provides an objective measure of VOR gain, a bedside version of the HIT (bHIT) provides a less sensitive (53), but still clinically useful indicator of normal vs. abnormal (overt refixation saccade = abnormal function) horizontal canal function (60). Agrawal et al. (61) showed that after controlling for age, older adults with a positive bHIT walked 0.23 m/s slower, had 0.44 additional falls in the past year, and were 5 times more likely to have fallen in the previous 5 years. Davalos-Bichara and Agrawal (62) went on to show that 100% of older adults (*n* = 15) with an abnormal bilateral bHIT were unable to sustain balance in condition 4 of the MRBT [similar to the results of Anson et al. (15) when using vHIT] (62). Also since bHIT is dependent upon observation of an overt re-fixation saccade, rather than a continuous measure of VOR gain, these correlations may be representative of a more severe loss of horizontal canal function. However, despite the diagnostic utility of VOR assessments, the VOR is rarely used during daily life without an accompanying input from the visual system; thus, measures of dynamic visual acuity have been developed as complementary measures of the “real world” function of the VOR, and by extension, the horizontal canals.

The Dynamic Visual Acuity Test (DVAT) (63) is a common method for measuring the functional (or real world) status of the VOR. The DVAT requires a subject to interpret the orientation of a letter whilst the head is rotated about an earth vertical axis at a constant velocity of 120–180°/s (64). DVAT has been shown to degrade with age (63, 65) and with peripheral vestibular loss (28, 29, 63), yet few studies have investigated the relationship between DVAT and imbalance among older adults without a known vestibular diagnosis. Hall et al. (25) observed that by performing exercises which target improvements in dynamic visual acuity (i.e., gaze stabilization exercises) both fall risk and dynamic balance were improved in a sample of older adults with non-specific, presumably age-related, dizziness. Using a similar test that measured visual acuity while walking on a treadmill, Lord and colleagues however found no relationship between dynamic visual acuity and measures of balance, falls, or gait among older adults (14, 66–69).

The Gaze Stabilization Test (GST) is a methodologically distinct, yet conceptually similar test of dynamic visual acuity that measures the maximum speed at which a subject can rotate their head while retaining the ability to perceive the correct orientation of a stable (i.e., fixed size) visual target (70). In a group of 86 healthy older adults, Ward et al. (71) showed that after correcting for age, deficits on the GST (performed in yaw) were associated with poorer performance on a series of eyes closed balance tasks. Honaker et al. (72) similarly showed that in a sample of older adults the GST was significantly correlated with the Dynamic Gait Index (DGI), a measure of dynamic, ambulatory balance (*r* = 0.65, *p* = 0.00009). Honaker et al. (72) also showed that the GST (using a cutoff velocity of 100.5°/s) was able to identify participants with a positive fall history and participants who were deemed to be at a heightened risk for falls based upon their DGI scores (Sensitivity: 0.80 and 0.79, Specificity: 0.87 and 0.81, respectively). Whitney et al. (73) however showed no relationship between the GST and scores on either the DGI or the timed up and go (TUG) in a control group consisting of 20 healthy older adults.

#### Otolith Organs

Cervical vestibular evoked myogenic potentials (cVEMPs) use an auditory tone burst to probe the portion of the descending vestibulospinal pathway between the saccule and the motor neurons in the cervical spinal cord (74, 75). McCaslin et al. (76) showed that increased cVEMP asymmetry was associated with greater postural sway on the sensory organization test (SOT); however, this finding was made in young adults (<65 years of age) with symptoms suggestive of vestibular pathology, limiting extension to the healthy aging population. In a sample of healthy (i.e., asymptomatic) older adults, Layman et al. (77) showed a significant relationship between gait speed, an indirect measure of postural stability (78), and the latency of cVEMPs. In the only study to our knowledge to compare balance and cVEMPs in healthy older adults, Anson et al. (15) showed that a bilateral absence of cVEMP responses was associated with a mild decrease in total sway area. The interpretation of this finding is unclear since the observed effect was opposite to what would be expected (i.e., increased sway area) if abnormal cVEMPs were indeed representative of the influence of otolith dysfunction on age-related imbalance; such findings may represent a guarded postural control strategy. The effect of cVEMP loss on postural sway was also substantially smaller than that of proprioception and was only significant when sway was measured during quiet stance on a firm surface with the eyes open (15), a condition where vestibular function is least likely to be required.

The proximal location of the measured myogenic response (the sternocleidomastoid) limits the interpretation of cVEMP abnormalities as evidence of age-related decline in the distal vestibulospinal pathways that mediate balance; this provides one potential explanation for the limited correlation observed between balance and cVEMPs. To address this limitation, Rudisill and Hain (79) investigated a novel variant of cVEMPs, the aptly named “leg-VEMP,” to probe the integrity of the more distal vestibulospinal pathways by recording EMG responses from the medial gastrocnemius. The primary limitation to measuring vestibular evoked myogenic potentials distally in a muscle that controls upright stance is the increased distance between the saccule and the targeted motor neurons. The added distance results in a lower amplitude and more variable EMG signal which limits any meaningful interpretation (79). Use of an intermediary muscle group, proximal to the MG but distal to the SCM (i.e., triceps brachii) (80, 81) has been found to produce a more robust response but yields little added benefit compared to traditional cVEMPs when the goal is to investigate the influence of saccular dysfunction on postural control.

### Electrical Stimulation of the Descending Vestibular Pathways

One limitation of cVEMPs is that auditory stimuli provide a rather weak stimulus to the vestibular labyrinth, with the intensity being limited by a risk of noise-induced damage to the middle ear. Galvanic vestibular stimulation (GVS) uses a direct electrical current applied to the skin overlying the mastoid processes to stimulate the vestibular afferents [predominantly irregular (82, 83)] that innervate both the otolith organs and semicircular canals (82, 84). In healthy adults, GVS causes a stereotyped response including postural sway toward the anode (i.e., away from the cathode) (85–87), ocular torsion (88–90), head and/or body tilt (91), an illusory sense of rotation (92, 93) or tilt (88), and an increase in EMG activity specifically in the muscles actively engaged in a postural task (e.g., soleus during upright stance) (94–96) [see (97, 98) for comprehensive reviews on GVS]. Studies that use GVS to probe descending vestibular control of balance typically measure either lower extremity EMG responses or the magnitude of GVS-induced postural sway (97). While well-characterized in healthy young adults (94) and subjects with vestibular pathology (85, 86), relatively few studies have investigated the responses to GVS in healthy older adults.

Welgampola and Colebatch (99) found a selective age-related change in the short latency GVS induced EMG responses in the soleus, such that the short latency responses in older adults were more delayed, more asymmetric, and lower in amplitude when compared to young adults. Dalton et al. (100) similarly found evidence of a selective decrease in the amplitude of short latency EMG signals in the soleus and medial gastrocnemius of older adults. Dalton et al. (100) also found a compensatory increase in tibialis anterior EMG responses to GVS, leading the authors to suggest a potential vestibular initiated compensation for age-related balance declines. Deshpande et al. (101) studied the influence of a combined stimulus including both GVS and a vibratory stimulus applied unilaterally to the dorsal aspect of the neck. They found that when walking with vision occluded, the young adult and older adult groups showed a similar increase in trunk velocity in the frontal plane. However, compared to young adults, the amount of lateral path deviation caused by the stimulus was attenuated in the older adult group, but this was only in regard to the response to the vibratory stimulus, not GVS (101). Tax et al. (86) however found that older adults showed a decrease in GVS induced postural sway during quiet stance. Yet, when compared to patients with bilateral vestibular hypofunction (86, 100), the reduction in GVS induced sway was minimal, potentially reflecting a more subtle decline in the descending vestibular pathways in older adults relative to patients with bilateral vestibular loss. Additional studies are needed determine if postural and EMG responses to GVS are predictive of balance impairment and fall risk in older adults.

### Balance Assessments

#### Measuring Sensory Contributions to Balance

The Romberg (RBT) and Sharpened Romberg (SRBT) balance tests determine an individual's ability to stand with a narrow base of support (RBT, feet together; SRBT, heel to toe) when vision is removed. Performance is determined by the subject's ability to maintain balance for a predetermined time interval (typically between 15 and 60 s) and is graded as success (able to remain upright) or failure (unable to remain upright). The insensitivity of the RBT to vestibular dysfunction prompted Fregly and Graybiel (102) to investigate the SRBT (and heel to toe walking) as a method to screen for vestibular disorders. Fregly and Graybiel (102) showed that individuals with the lowest caloric responses showed the greatest deficits in both the SRBT and heel to toe walking when the eyes were closed. However, more recently, in an older adult population, Longridge et al. (103) showed that the SRBT was unable to separate healthy older adults from those with known vestibulopathy, with most subjects falling regardless of the presence of vestibular pathology; such differences from the original findings of Fregly and Graybiel (102) are likely explained by an age-associated floor effect of the SRBT due to the high degree of challenge. Such limitations provide support for the use of more comprehensive tests when attempting to detect vestibular contributions to imbalance.

One of the most frequently used methods is computerized dynamic posturography (CDP), and more specifically the sensory organization test (SOT) (36, 104–106). The SOT uses a sophisticated motion platform to quantify changes in a patient's postural sway (i.e., movement of the center of pressure, CoP) when the fidelity of visual and/or somatosensory cues is altered (105, 106). Pairing movement of either the visual surround and/or the surface with the participant's postural sway (i.e., sway referencing) diminishes one's ability to use proprioceptive and/or visual feedback for postural control. Therefore, the magnitude of postural sway is dependent upon the individual's ability to suppress any conflicting visual and/or proprioceptive cues and weight the remaining veridical cues (107). The vestibular system is the only unperturbed sensory modality controlling upright stance in conditions five and six of the SOT (SOT-5, SOT-6; Table 1), where proprioception is unreliable, and vision is either unreliable (SOT-6) or removed (SOT-5). As a result, patients with an acute vestibular lesion demonstrate marked instability in these conditions (111–115) and performance on SOT-5 and SOT-6 (relative unperturbed quiet stance, SOT-1) has been adopted as a surrogate measure of vestibulospinal function.

**Table 1:** Table consists of conditions for the SOT, including parameters for head-shake variations established by both Mishra et al. (108) and Honaker et al. (109), respectively.

**Condition**	**Vision**	**Platform**	**Unperturbed sensory systems**	**Head shake variation**
SOT-1	Eyes Open + Stationary Surround	Stationary	Visual; Somatosensory; Vestibular	
SOT-2	Eyes CLOSED	Stationary	Somatosensory; Vestibular	60°/s (108)
				120°/s (109)
SOT-3	Eyes Open + Sway Referenced	Stationary	Somatosensory; Vestibular	
SOT-4	Eyes Open + Stationary Surround	Sway-Referenced	Visual; Vestibular	
SOT-5	Eyes Closed + Stationary Surround	Sway-Referenced	Vestibular	15°/s (109)
				60°/s (108)
SOT-6	Eyes Open + Sway Referenced	Sway-Referenced	Vestibular	

*Graphical depictions for each condition can be found at (108–110)*.

Evidence supporting use of SOT to detect *age-related* vestibular declines is not as straight forward. The output of the SOT, postural sway, is a complex sensorimotor response that requires the integration and weighting of multiple sensory cues and a sensorimotor transformation that ultimately produces a response using the distal musculature (116). Older adults experience an increase in reaction times (117), a decline in lower extremity strength (20), and reduced distal somatosensation (14, 16, 118); each of which can potentially influence balance (118). As a result, postural sway in SOT-5 and SOT-6 cannot with certainty be attributed solely to an age-related decline in vestibular function. This is highlighted by impaired performance in SOT-5 and SOT-6 (i.e., the “vestibular conditions”), in both healthy adults (119, 120) and in patients with various central nervous system pathologies (121), two populations without vestibular impairment.

Consistent with this notion, studies using SOT-5 and SOT-6 to probe vestibular contributions to age-related imbalance have produced conflicting results over the past 30 years (122). Woollacott et al. (123) showed that older adults were more likely to fall during SOT-5 rather than SOT-6, prompting the authors to propose a pattern of imbalance consistent with dysfunction in the vestibular periphery. Similarly, Ledin et al. (124) showed that sway was significantly increased in SOT 5, but not SOT-6, when comparing participants 70–75 years of age to participants 60–69 years of age. However, several authors have since identified a different pattern of imbalance among older adults, consistent with an age-related deficiency in the ability to resolve sensory conflict (46, 119, 122, 125–128), rather than a loss of peripheral vestibular inputs. Whipple et al. (122) and Wolfson et al. (126) each showed that when compared to young adults, older adults showed a larger increase in sway and a greater prevalence of falls, in conditions with inaccurate (SOT-6), rather than absent (SOT-5), visual feedback. Peterka and Black (36), observed that older adults fell most often when experiencing conflicting visual and proprioceptive cues (SOT-6), and when a fall did not occur, sway was increased in SOT-6 relative to SOT-5. Interestingly, the older adults in Peterka and Black's study that were able to remain upright for SOT-5 showed similar amounts of sway to the young adult control group (125). More recently, Pedalini et al. (127) showed that while older adults with symptoms of vestibular impairment performed worse on SOT-4–6 than asymptomatic older adults, asymptomatic adults also performed significantly worse than young adults in each of these same conditions. Thus, instability in SOT conditions 4–6 may be sensitive to aging, independent of vestibular decline. These findings lend support to the general conclusion that the instability observed in the vestibular conditions of the SOT may be indicative of an age-related inability to suppress conflicting sensory cues (107), suggesting a breakdown in the central integration (36, 122, 126) or reweighting of multiple sensory cues, both vestibular and extra-vestibular, rather than direct evidence of impaired descending vestibular function.

Furthermore, even in subjects with a known vestibular lesion, the sensitivity of the SOT to vestibular loss has been shown to be only around 50% (109, 121, 129). In an effort to improve sensitivity, Mishra et al. (108) studied the effect of adding active yaw head rotations to SOT-2 and SOT-5 (Table 1). Yet, the aptly named head shake SOT (HS-SOT) was found to offer little benefit over the traditional SOT secondary to a ceiling effect in HS-SOT-2 and a floor effect in HS-SOT-5 (108). Park et al. (130) tested the HS-SOT in a sample of 102 healthy older adults. The ratio between SOT-5 and HS-SOT-5 was found to be 54% higher in older adults compared to young adults; the authors concluded that the increase in sway with the addition of the head shake stimulus was indicative of impaired vestibular function in the older adult group (130). However, as stated in the original study by Mishra et al. (108), the HS-SOT-5 is exceptionally challenging, such that subjects with a known history of vestibular hypofunction could not be differentiated from healthy adults due to universally poor performance. Thus, the increased instability in HS-SOT-5 compared to SOT-5 seen by Park et al. (130) may simply reflect the added challenge, rather than the presence of vestibular impairment. Honaker et al. (131) has since addressed this floor effect by decreasing the rate of head rotation in SOT-5 (Table 1), improving upon the sensitivity (70%) of HS-SOT for unilateral vestibular loss (109); future studies should consider investigating this paradigm in an older adult population.

The clinical test of sensory interaction and balance (CTSIB) is a simplified version of the SOT, developed specifically for use in a clinical setting (13, 132). Subjects perform tasks analogous to the six conditions of the SOT, with the exception that sway referenced supports are replaced by foam and sway referenced visual surrounds are replaced by an opaque globe, meant to distort but not remove visual inputs (132). CTSIB conditions are scored based on the amount of time one can maintain upright stance, up to a maximum of 30 s, in each condition (132). Cohen et al. (133) investigated the use of the CTSIB in patients with vestibular disorders compared to older adults. Cohen et al. (133) showed that conditions 4 and 6, but not condition 5, of the CTSIB were more affected by vestibular dysfunction than by healthy aging, suggesting a potential role for conditions 4 (globe plus firm surface) and 6 (globe plus foam surface) as a screening tool for vestibular dysfunction in older adults. Many clinicians and researchers have however opted for the use of a further simplified test, the modified CTSIB (mCTSIB), which consists only of static stance on both firm and foam surfaces, with the eyes open or closed (134, 135).

Condition 4 of the mCTSIB (i.e., condition 5 of the CTSIB) requires the participant to stand with the eyes closed, atop a foam pad meant to degrade somatosensory feedback from the distal lower extremity, similar to the sway referenced conditions of the SOT. Hong et al. (136) showed that although the full mCTSIB was a poor predictor of performance on the SOT, condition 4 of the mCTSIB correlated with SOT-5, demonstrating validity of this relatively simple test as an appropriate method for degrading somatosensory feedback. Since closing the eyes also removes visual input, leaving the vestibular system as the only undisturbed contributor to upright stance, an inability to maintain balance in condition 4 of the mCTSIB has been proposed as an indicator of impaired vestibulospinal function. An analogous test [a modified Romberg Balance Test (MRBT)], was recently used in a nationally representative sample of older adults, the National Health and Nutrition Evaluation Survey (NHANES), as a screening tool to estimate vestibular impairment (23). Agrawal et al. (23) reported that 34.5% of adults over the age of 40 and more than half of adults over age 60 failed condition 4 of the MRBT (i.e., could not stand 20 s on foam with eyes closed) with failure being associated with over a 6-fold increase in the odds of having experienced a fall in the previous year (23). The authors suggested that such findings represent the burden of vestibular dysfunction among older Americans (23). This suggestion has however been subsequently challenged. As described above, Jacobson et al. (41) found weak correlations between vestibular function tests and failure on the MRBT in a sample of adults over the age of 40, drawing the conclusion that the MRBT may be inappropriate as a screening tool for vestibular impairment in older adults. However, poor agreement between posturography and vestibular function tests need not discredit the applicability of such assessments, as one should expect dissimilar findings when using tests which probe different vestibular function contributions.

#### Static Posturography and Computational Approaches

As an alternative to the SOT assessment and the simpler RBT, sRBT, and mCTSIB assessments, a potential middle ground exists using digitized posturography tools, such as force plates, pressure sensors, and inertial measurement units, to track movement of the CoP during quiet stance. Such an approach improves upon the sensitivity of the time to failure scoring of traditional clinical balance tests (137) and takes less time to administer than the complete set of conditions performed in the SOT. Baloh et al. (138) used force plates to measure the velocity of postural sway in conditions analogous to the mCTSIB. Baloh found that older adults with a history of imbalance demonstrated an increase in sway velocity, regardless of the presence of vestibular hypofunction, when standing on foam with the eyes closed (138). Therefore, a critical consideration to making posturography during quiet stance valuable as a vestibular assessment is whether computational approaches can extract characteristics of the CoP motion that discriminate between those with vestibular hypofunction and those with normal vestibular function.

Recently, Gilfriche et al. (139) analyzed CoP data using a modified version of detrended fluctuation analysis (140), Frequency Specific Fractal Analysis (FsFA), which converts time scales (N) to frequency spectra (Fc) (Fc = Sample Frequency/*N*) (141). Gilfriche found that performing a fatiguing lower body exercise influenced the scaling exponent that described CoP fluctuations at higher frequencies (2–20 Hz), a frequency range thought to be dominated by somatosensory control, with no change in the lower frequency scaling exponent (<0.5 Hz), a frequency range suggested by Gilfriche to reflect visuo-vestibular control (139). Yet, such assumptions are in opposition to the preferential sensitivity of the vestibular afferents to high, rather than low, frequency stimuli (141). Consistent with this opposing view, using empirical mode decomposition, Yeh et al. (142) showed that young adults with vestibular disorders could be differentiated from healthy older adults by a characteristic increase in power within the high frequency range of the CoP time series. Thus, while these computationally driven analyses of quiet stance appear to be sensitive to both physiologic changes (i.e., somatosensory loss) and the effects of aging, the capacity to use these methods to detect alterations in postural sway that result specifically from age-related vestibular decline is still largely unknown. Future analysis of CoP complexity may benefit from 1) analyzing the dynamics of postural sway using SOT-5, condition 4 of the mCTSIB/MRBT (see above), or pseudorandom perturbations (see below), rather than unperturbed quiet stance and 2) by determining the association between the CoP frequency spectra and objective measures of sensory precision (e.g., perceptual thresholds).

#### Pseudorandom Perturbations

Peterka (37) developed a closed loop model of postural control to describe sagittal plane control of the center of mass when presented with ambiguous or unreliable sensory cues. In the model, postural sway results from an active corrective torque at the ankle proportional to the relative weighting of visual, proprioceptive, and vestibular cues (37). To empirically test the validity of this closed loop model, subjects were exposed to continuous rotations of the support platform or visual surround using a pseudorandom sequence of summed sinusoids while in upright stance. The perturbations were delivered under various sensory conditions (Table 2), including a sway-referenced platform or visual surround (as is done in the SOT). The use of external perturbations allows for the calculation of transfer functions that quantify the response of the postural control system as a function of the frequency spectra and amplitude of the imposed platform perturbation (37, 143). Peterka compared the gain and phase (i.e., the magnitude and timing of sway relative to the platform, respectively) of postural sway derived from these transfer functions to compare the behavior of healthy young adults to those with compensated bilateral vestibular loss (37). Peterka showed that when only vestibular and proprioceptive cues were available (i.e., with eyes closed) patients with compensated unilateral (UVL) or bilateral vestibular loss (BVL) adopted a distinct pattern of sway in response to anteroposterior tilts of the platform (37, 144). At large amplitudes of tilt, it becomes advantageous to realign the body with gravitational vertical, rather than the platform; these participants however showed a monotonic increase in postural sway at increasing angles of platform tilt, reflecting a tendency to continue to align the center of mass with the support surface (37, 144). In the context of the closed loop model of postural control, this pattern can be interpreted as a persistent reliance upon proprioceptive feedback or a deficiency in the reweighting of the absent (BVL) or impaired (UVL) vestibular cues; this is highlighted by the comparison to healthy adults who instead readily reweight the intact vestibular cue, aligning the body with gravitational vertical rather than the platform during larger amplitude perturbations (37, 144).

**Table 2:** Conditions of the protocol developd by Peterka (37) using pseudorandom perturbations to model postural sway.

**Condition**	**Vision**	**Platform**	**Sensory information**		
			**Vision**	**Proprioception**	**Vestibular**
1	PRTS stimulus	Fixed	Accurate	Veridical	Veridical
2	PRTS stimulus	Sway-referenced	Accurate	Inaccurate	Veridical
3	Fixed	PRTS stimulus	Veridical	Accurate	Veridical
4	Eyes closed	PRTS stimulus	Absent	Accurate	Veridical
5	Sway-referenced	PRTS stimulus	Inaccurate	Accurate	Veridical
6	PRTS stimulus	PRTS stimulus	Accurate	Accurate	Veridical

*Recreated from Peterka (37). PRTS, pseudorandom ternary sequence*.

The aforementioned studies (37, 144) included only patients who were more than a year removed from their injury and therefore were believed to be well-compensated (145); this highlights the sensitivity of this method to subtle vestibular declines, such as occur with aging (34). Cenciarini et al. (146) used pseudorandom platform tilts in a sample of healthy older adult subjects and showed that, relative to young adults, older adults showed a compensatory increase in stiffness and damping parameters, interpreted by the authors as evidence that older adults adopt a unique compensatory strategy to maintain balance (146). Unfortunately investigating the weighting of vestibular and proprioceptive cues was not a goal of their experiment. Wiesmeir et al. (147) showed that unlike patients with vestibular loss, healthy older adults showed a retained capacity to increase the use of vestibular cues at higher amplitudes of platform tilt. Yet, they also found that relative to young adults, older adults displayed a consistently increased weighting of proprioceptive cues. Since the group of older adults were asymptomatic and had normal VOR function on rotational testing, the observed age-related changes in sensory reweighting mechanisms may be related to subclinical declines in vestibular pathways distinct from those mediating the VOR. Yet, the relevant vestibular structure (i.e., otolith, canal, or central) underlying the ability to orient one's self with gravitational vertical during dynamic pseudorandom perturbations remains unknown.

#### Standardized Measures of Functional Mobility

The Fukuda (i.e., Unterburger) Stepping Test is a classic bedside test designed to assess descending vestibulospinal control by measuring the degree of rotation that occurs while stepping in place with the eyes closed (148). Studies showing poor sensitivity and specificity of the Fukuda Stepping Test for vestibular dysfunction (131, 149) have led many to adopt use of more comprehensive functional assessments. Several standardized tests incorporate elements meant explicitly to perturb the vestibular system (e.g., rotating the head and/or body) to determine a vestibular cause to postural instability. The timed up and go (TUG), DGI, and functional gait assessment (FGA) measure dynamic ambulatory balance and are capable of predicting fall risk among older adults (150–152) and patients with vestibular loss (153, 154). The DGI and FGA require subjects to ambulate while rotating their head in either pitch or yaw (151, 155), whereas the TUG requires subjects to walk a distance and then turn the body 180 degrees while walking (154). Although individuals with vestibular impairment struggle with the aforementioned tasks (154, 156, 157), it is worth emphasizing that the vestibular system is not the sole respondent to the imposed stimuli. Chang and Schubert (158) showed that DGI scores improved independent of VOR gain improvement in a sample of patients recovering from unilateral vestibular deafferentation surgery. Actively rotating the head involves vestibular, cervical proprioceptive, motor efferent, and visual cues; a compensatory change to any of these extra-vestibular systems could therefore improve balance performance independent of vestibular loss or recovery. Thus, imbalance provoked by movement of the head, while certainly a symptom of suboptimal vestibular function, cannot definitively indicate vestibular loss. Cohen et al. (134) did however find that the addition of yaw and pitch head movements improved the sensitivity of eyes closed heel to toe walking and the mCTSIB to vestibular pathology (e.g., Meniere's disease, BPPV) in older adults aged 40–79; whether this finding is generalizable specifically to sub-clinical vestibular declines, rather than the identification of vestibular pathology, remains to be seen.

Overall, functional outcome measures allow clinicians to efficiently predict fall risk and are often used to guide rehabilitative efforts (152, 155, 159). The capacity to mimic real world balance perturbations (e.g., stepping over an object) likely explains their predictive abilities. However, this functional emphasis conversely prevents such tests from isolating the culpable system causing the balance disturbance and this is particularly true in the evaluation of the older adult patient, as imbalance on clinical balance tests can be caused by a number of extra-vestibular age-related changes in sensorimotor function (e.g., kinesthesia, strength) (160).

### Perception of Subjective Vertical

#### Subjective Visual Vertical

Tests of subjective visual vertical (SVV) quantify the relative contributions of otolith (predominantly utricle), visual, and somatosensory cues by estimating an individual's perception of gravitational vertical [See (161) for a review]. While methods vary, SVV generally requires an individual to orient an illuminated bar with what they perceive to be true vertical; testing can take place either in the dark (i.e., no veridical visual cues) or in the presence of a moving (dynamic SVV) or inaccurate (rod-and-frame SVV) visual cue. Tobis et al. (162) found that older adult fallers showed a significant bias in SVV in the direction of a tilted frame that surrounded the vertical bar (i.e., rod-and-frame SVV), suggesting an increased weighting of visual, relative to otolith cues, presumably as a compensation for vestibular declines (161). Lord and Webster (163) showed that when compared to healthy older adults, older adults with a positive fall history displayed greater errors (deviation from true vertical) in both static (fixed background) and dynamic (rotating background) SVV tasks. Barr et al. (164) showed that when in the presence of a rotating visual surround, a deviation in SVV of at least 6.5° was associated with greater fall risk and a significant decrease in postural sway when standing with the eyes open or closed, and when standing in the presence of a moving visual field; such patterns suggest the adoption of a maladaptive stiffening of one's postural control strategy. Together, these results suggest that changes in the weighting of otolith relative to visual cues may predict altered postural control strategies and fall risk in older adults.

The presence of a stereotyped bias in SVV toward the side of an acute unilateral vestibular lesion suggests that the otolith organs are at least one of the principle contributors to one's estimate of subjective vertical (165). Yet, SVV is restored within weeks to months after a vestibular lesion (166, 167). This suggests that an individual with persistent vestibular loss can restore SVV by appropriately weighting residual vestibuar cues with intact visual, somatosensory (168) and/or somatic graviceptive (e.g., vascular receptors signaling a shift in blood volume within the trunk) cues (169). Also, it appears that a maladaptive reweighting of verticality cues, rather than isolated vestibular loss, may explain the correlations observed between SVV, fall risk, and postural sway in older adults, as such relationships are observed when SVV is measured in the presence of a spurious visual reference (i.e., tilted frame or rotating background) (162–164).

#### Subjective Postural Vertical

SPV is typically measured by having an individual report when they perceive their body to be aligned with gravitational vertical. SPV can be quantified both by the accuracy (deviation relative to upright) and precision (variability around the perception of upright) of their estimate of vertical (161). Barbierri et al. (170) showed that SPV gradually tilts backward with age, with nearly a doubling of backward inclination in healthy adults over 50 (−1.15° ± 1.40) when compared to adults 18–49 years of age (−0.45° ± 0.97, *p* < 0.01). Manckoundia et al. (171) found that this age-associated posterior displacement of the SPV was highly correlated (*r* = 0.95, *p* < 0.000001) with the extent of backward instability during a series of balance and mobility tasks (e.g., sitting, standing with and without vision and transitioning between sitting and standing). This deviation of SPV among older adults has been posited as a potential explanation for an asymmetrical pattern of postural instability experienced by older adults referred to as “backward disequilibrium” (170–172). Menant et al. (173) showed that both the precision and accuracy of SPV in the mediolateral direction was also significantly correlated with lateral sway (*r* = 0.20 and 0.16, respectively) measured during stance in a foam, eyes closed condition. This relationship persisted after correcting for age, gender, and other general indicators of mobility (i.e., the physiological profile assessment); considering the large sample size (*N* = 195), this finding provides convincing evidence that perceived vertical may be a potentially useful indicator of age-related balance dysfunction.

Yet, Bisdorf et al. (165) showed that neither chronic nor acute vestibular lesions were sufficient to induce a bias in SPV. Anastopolous similarly showed that SPV remained veridical following an acute vestibular lesion, despite a consistent tilt of SVV toward the side of the vestibular lesion (174). Ito and Gresty (175) showed that even in individuals with a total surgical loss of vestibular function (due to surgery to correct Neurofibromatosis Type II), the accuracy of SPV remained similar to healthy adults. These studies suggest that unlike SVV, otolith dysfunction has minimal impact on the accuracy (i.e., bias) of SPV. Instead, the increased variability of SPV in patients with bilateral vestibular loss and acute unilateral lesions suggests that graviceptive otolith cues may instead be more relevant for reducing the variability in one's estimate of postural vertical (161, 165). A clear bias in SPV has however been observed in individuals with somatosensory loss affecting the trunk (176, 177). Thus, the association between imbalance and the observed age-related shift in SPV, may be indicative of the impact of age-related somatosensory declines or alterations in the central integration of somatic graviceptive, somatosensory, and vestibular cues.

### Vestibular Noise

#### Vestibular Perceptual Thresholds

Neural noise (referred to herein as “noise”) is the random fluctuation in neural activity that occurs within each sensory system (178, 179). While low levels of external noise can enhance sensory signals [i.e., stochastic resonance (180)], excess internal noise impairs the communication between neurons, and has been posited as a potential cause of age-related cognitive decline (178, 181, 182). Neurophysiologic studies have provided evidence for age-related increases in neural noise, showing a relationship between electroencephalogram (EEG) 1/*f* power spectral density and both language and visual working memory in older adults (182). Behavioral studies have similarly shown that background sensory noise impairs the perception of visual images in older adults (178). Psychophysical assessments of self-motion perception (“vestibular perceptual thresholds”) provide evidence that neural noise may also affect the aging vestibular system (22, 183, 184).

Consistent with classic signal detection theory (185), a *vestibular threshold* represents the minimum stimulus value at which a subject can accurately (at a pre-defined accuracy level) perceive the direction of passive self-motion while using predominantly vestibular cues (186). In other words, a perceptual threshold is the magnitude of a vestibular stimulus required to overcome the level of internal noise within an individual's vestibular system, thereby allowing the self-motion signal to be reliably perceived by the subject (e.g., “I moved right”); *thus, vestibular thresholds are an assay of vestibular noise* (186). Although methods may vary, one-interval direction recognition tasks (e.g., “did I move right or left?”) are the most common psychophysical technique for quantifying vestibular perceptual thresholds (186). By quantifying vestibular noise during self-motion, vestibular thresholds can independently assess each vestibular modality (tilt, rotation, and translation). While a comprehensive review of vestibular perceptual testing is beyond the scope of this review, we refer the interested reader to several reviews on the topics of signal detection theory and vestibular perceptual thresholds (185–189).

#### Vestibular Thresholds and Age-Related Imbalance

Vestibular thresholds have been shown to increase after age 40 in all planes of motion including supero-inferior translations, interaural translations, roll tilt, and yaw rotation (22). Despite thresholds being moderately correlated with one another, only low frequency (0.2 Hz or 5 s per motion) roll tilt perceptual thresholds show a significant association with *age-related* imbalance (190). Elevated roll tilt thresholds were found to be associated with an increase in the likelihood of failing (i.e., falling prior to the end of the 30 s trial) the “eyes closed, standing on a compliant surface” test condition of the MRBT in a sample of healthy adults over the age of 40 (190); this statistically significant relationship remained even after accounting for age. In a subsequent mediation analysis of these data, Beylergil et al. (17) showed that roll-tilt thresholds significantly mediated the relationship between age and balance impairment. Despite the multitude of factors that contribute to age-related balance impairment (e.g., vision, cognition, kinesthesia), this analysis showed that age-related increases in roll tilt thresholds accounted for nearly half (46%) of the relationship between age and balance impairment (17). We posit three possible explanations for this admittedly surprising finding, (1) roll tilt perceptual thresholds may represent a physiologically relevant vestibular cue (190) due to the inherent need for human beings to respond to changes in head position relative to gravity (i.e., tilt) in order to remain upright, (2) roll tilt perceptual thresholds may reflect a more global measure of central vestibular function, as the precision of roll tilt perception is dependent upon the ability of the aging brain to perform (191–197) complex neural computations to resolve ambiguous gravitoinertial cues from the otolith organs, and (3) canal and otolith cues are integrated during most, if not all, naturalistic human motions (198), and thus impaired roll tilt perception may specifically reflect imperfections associated with canal-otolith integration. Yet, similar to vHIT, such comparisons have been restricted to balance performance in quiet stance on a foam pad with the eyes closed, and thus one must be cautious with the extrapolation of these findings to alternative aspects of human balance.

#### Noise, Aging, and Velocity Storage

Recent empirical and modeling efforts have further implicated noise as an explanation for age-related changes in the temporal characteristics of the VOR (199) and in age-related changes in postural control (200). During a sustained, constant velocity yaw rotation about an earth vertical axis, activity in the peripheral vestibular afferents degrades quickly, with a time constant of around 4–6 s (141, 201, 202). However, compensatory nystagmus and the subjective perception of rotation persist up to 30 s (34, 43, 203); this propagation of behavioral vestibular activity is accomplished through a mechanism called velocity storage [see (204) for a review on this topic but also see (205, 206) for differing perspectives]. A reduced time constant (i.e., faster decay of nystagmus) has been identified in subjects with unilateral and bilateral vestibular loss (207), in those with central vestibular dysfunction (208), and in asymptomatic older adults (34, 35, 203, 209).

Karmali proposed an explanation for the observed effect of aging on the vestibular time constant (Tc) using a Bayesian framework (199, 210–212). Extending the Tc beyond the time constant of peripheral vestibular afferents requires the repeated integration of canal signals (213) which amplifies the accompanying noise (199). Thus, while prolonging the Tc results in a more accurate estimate of rotation, it comes at the cost of an accumulation of noise (i.e., reducing precision) (212). Using a Bayesian optimal Kalman filter model, Karmali et al. (199) showed that the age-related shortening of the Tc may be proportional to published data describing the rate of age-related decline in the vestibular hair cells (214). Karmali suggested that in order to compensate for this decline in the vestibular signal to noise ratio, the CNS may dynamically shorten the Tc to achieve an optimal balance between accuracy and precision (199, 212).

While vestibular noise, assayed by roll tilt perceptual thresholds, has been found to be associated with age-related balance impairment, similar work comparing the Tc to measures of balance is sparse. In the only study to our knowledge to empirically compare the Tc and balance, Jacobson et al. (215) showed that a lower Tc, even in the presence of normal VOR gain, was associated with greater postural instability among otherwise healthy older adults. It is worth noting that concomitant ischemic pontine lesions were also identified in ~80% of the older adults with a balance impairment and shortened Tc, suggesting that velocity storage may be reflective of central vestibular dysfunction in older adults (215). Additional work is needed to determine if the Tc represents the contribution of central vestibular pathways to age-related balance declines.

## Discussion

Measures of low frequency horizontal canal function (i.e., caloric irrigation and rotational chair testing) appear to provide minimal benefit when attempting to identify a vestibular cause to age-related imbalance. Alternative measures of canal function that include the integration of visual cues (i.e., GST) or a high frequency stimulus (i.e., vHIT), have instead demonstrated a capacity to predict postural instability and fall risk in older adults (71, 216). Given that correlations remained even after correcting for age, these findings indicate that vHIT and GST may be suitable measures to quantify specific contributions of the horizontal canal to age-related imbalance. However, one must consider several limitations when relying on assessments of the VOR to quantify the influence of impaired canal function on age-related imbalance.

Measures of the VOR are not solely measures of canal function, but instead reflect the integrity of the motor limb of the reflex (e.g., oculomotor plant) and can be influenced by extra-vestibular oculomotor strategies (e.g., compensatory saccades) (64, 217, 218). This point is highlighted by the typical course of recovery following unilateral vestibular loss (UVD). Dynamic visual acuity improves independent of any restoration in peripheral vestibular function (26, 27, 145, 219) secondary to the recruitment of compensatory saccades (220), the unmasking of cervical proprioceptive inputs (221–224) and the integration of motor efferent signals (64, 217, 218). Also, while the horizontal canals feed into both the ascending and descending vestibular pathways, VOR pathways are anatomically distinct from the vestibulospinal pathways (222, 223), diverging at the level of second order neurons in the vestibular nuclei [VOR via position vestibular pause (PVP) and vestibulospinal via vestibular only (VO) neurons, respectively; see (225) for a review on this topic]. Thus, the association between VOR measures and imbalance may be representative of imbalance that results from unstable gaze (the function of the VOR), rather than as an indication that such measures directly assay the pathway connecting the horizontal canals to the descending vestibular pathways. Nevertheless, VOR assessments irrefutably remain vital to lateralize vestibular loss (226, 227).

Since balance assessments, namely the SOT, directly measure balance performance, in principle, they are likely considered better suited to identify imbalance that results from vestibular impairment. The vestibular conditions of the SOT (i.e., SOT-5 and 6) have however shown limited sensitivity (109, 121, 129) and specificity (119–121) when used as a diagnostic site of lesion test. This is likely a result of the inability to differentiate a patient with restored vestibular function from a patient who has successfully re-weighted extra-vestibular cues and the analytical consideration of the “vestibular sense” as a single sense, when, in fact, it includes at least 3 sensory modalities (rotation, translation, tilt) (228, 229). These issues are accentuated when examining older adults due to (1) the inability to separate dysfunction of the descending vestibular pathways from alternative age-associated sensorimotor declines, (2) the relative insensitivity to subtle changes in vestibular function, and (3) the fact that all vestibular modalities, that may decline at different rates with aging, are lumped into a single entity. This is not meant to dissuade clinicians and researchers from using the SOT when vestibular decline is suspected, but rather to suggest that it be used as a vestibular screening tool and/or be paired with more direct assays of vestibular function. Asai et al. (230) showed that sensitivity of the SOT was improved when considered in conjunction with positive findings on a standard vestibular test battery. Cohen et al., recently showed that combining SOT-5 with a clinical balance test [e.g., the functional mobility test (FMT), Timed Up and Go (TUG), or Dynamic Gait Index (DGI)] improved sensitivity to vestibular impairment in a group of young adults (231).

Some of these limitations of the SOT may be addressable by treating postural sway as the output of a closed loop system, rather than directly as evidence of instability. In the SOT, as is true in nearly all balance tests, increased movement of the CoP is interpreted as evidence of instability. However, considering the magnitude of postural sway in isolation does not in of itself indicate instability, and may instead represent exploratory behavior (232) or could be the result of a compensatory re-weighting of sensory cues (37). By measuring sway evoked by continuous pseudorandom perturbations, one can compute transfer functions which determine if the center of mass orients relative to the stimulus (i.e., tilting platform), suggesting persistent weighting of proprioceptive cues, or with gravitational vertical, suggesting an amplitude dependent reweighting of vestibular cues (37, 233); this approach provides a quantitative explanation for the observed postural sway (37). Thus, in experimental conditions that alter the reliability of sensory cues (i.e., eyes closed or sway referencing) such an approach may offer improvements on the SOT by determining the postural control strategy being used to respond to the externally imposed perturbations and the relative weights assigned to each sensory channel. Pseudorandom stimuli also have the advantage of providing a less predictable perturbation to the vestibular system, an approach previously shown to be more discriminatory for identifying vestibular pathology (217).

Yet, an inherent limitation to the use of any complex sensorimotor task, including the SOT or pseudorandom perturbations, is that the output (i.e., sway as measured using CoP or CoM) is the only quantifiable indicator of performance. As a result, if one is attempting to determine if vestibular *sensory* function is the cause of a patient's imbalance or falls, this can only be *indirectly* inferred from the pattern of *motor* outputs. Similar to compensatory adjustments of the VOR by the oculomotor limb of the VOR, the results of posturographic assessments must be considered in light of potential downstream alterations in the motor limb of the vestibulospinal reflexes. In an oversimplified example, consider for a moment the optimal output of the vestibulospinal reflex in response to a perturbation, such as a sudden translation of the head. If the vestibular afferents, due to an age-related loss of hair cells (214), trigger a motor response that compensates for only 80% of the perturbation, the descending motor system could (in theory) simply boost its gain to 120% and the net output of postural sway will mirror a system with normal vestibular function. Redundancies and compensatory mechanisms, while beneficial to maintain mobility and prevent falls, can make isolating the cause of balance disturbance a daunting task; such limitations motivate attempts to directly stimulate the vestibular labyrinth.

Beyond head motion, GVS may provide the most direct method for measuring the descending vestibular reflexes. The interpretation of GVS is however bounded by the dependence of the motor outputs on the context of the experimental task (234). Width of the base of support (91, 235), upper extremity support (96), availability of vision (96, 235, 236), reliability of proprioceptive feedback (234, 235), and testing posture (sitting vs. standing) (91, 96) have each been shown to modulate the magnitude of GVS induced EMG and/or postural responses, and thus can result in attenuated or enhanced responses to GVS, *independent of the integrity of the descending vestibular pathways*. The use of GVS paradigms in older adults can therefore only be interpreted relative to the reliability of extra-vestibular sensory cues [somatosensory (236, 237) and visual (234)] which are known to also decline with age (14, 118, 238, 239). Comparing responses to GVS in healthy older adults to older adults with vestibular decline, using tightly controlled experimental conditions that control for age-related visual and somatosensory loss, could however yield an interesting avenue for future research.

The limitations of existing assessments have motivated our recent study of vestibular perceptual thresholds as an assay of vestibular noise (17, 22, 190). As mentioned above, research out of our lab identified a significant association between 0.2 Hz roll tilt perceptual thresholds and age-related imbalance, yet, we emphasize that we are not suggesting that an inability to perceive roll-tilt is the cause of age-related balance impairment. We instead put forward the hypothesis that age-related changes in roll tilt vestibular thresholds reflect central vestibular noise that affects both perception and balance and thus may provide a method to quantify contributions of the central vestibular system to age-related balance impairment. The significant mediating effect of roll tilt thresholds and balance may therefore implicate central vestibular noise as a principle contributor to age-related imbalance. Considering that the participants studied were without symptoms of vestibular impairment, the observed age-related increase in vestibular thresholds also highlights the potential impact of sub-threshold (i.e., below the threshold for triggering symptoms) vestibular noise on age-related imbalance.

Although speculative, one potential explanation for this association is an age-related increase in noise within a specialized class of neurons within the vestibular nuclei [vestibular-only (VO) neurons] that act as relays for both vestibulospinal control and self-motion perception [see (240) for a review]. As justification for this hypothesis, the VOR and self-motion perceptual pathways relay through different neurons in the VN, and as such show qualitatively dissimilar behavior (193, 194). As an alternative explanation, since the perception of tilt is dependent upon one's ability to use vertical canal cues to disambiguate changes in net gravitoinertial force sensed by the otoliths (195), roll tilt thresholds may instead more generally represent a measure of age-related changes in central canal-otolith integration. Wiesmeir showed that postural responses to pseudorandom tilts of the support surface differed significantly between young and older adults within the frequency range (0.15–0.40 Hz) (147) where canal and otolith cues have been shown to be optimally integrated when rotating about an earth horizontal axis (241). Similar performance between age groups at 0.05 Hz suggests that at frequencies below the threshold for vertical SCC activation, where otolith cues predominate, the gain of postural sway is instead unaffected by age (241). Such findings lend support to our hypothesis that an age-associated increase in central vestibular noise, manifesting as a breakdown in central canal-otolith integration, may serve as a principle contributor to age-related balance declines.

While such explanations are plausible, as of now empirical proof is lacking, thus it does remain possible that roll tilt perceptual thresholds and balance are simply parallel yet independent consequences of aging. However as previously stated by Karmali et al. (190), this alternative is unlikely given that one would expect that if a common central nervous system pathology caused the correlation between roll tilt thresholds and balance, that most, if not all, vestibular thresholds, not just 0.2 Hz roll tilt, would display this same predictive relationship. Nevertheless, correlation does not equal causation, therefore additional studies are needed to (1) determine if the relationship between vestibular noise and age-related balance impairment is indeed causal, (2) to confirm or refute these findings in an independent sample of older adults, and (3) determine the mechanism explaining the association. Determining if alternative measures of vestibular noise, such as the vestibular time constant (199, 212) or measures of VOR variability (242), display similar associations with age-related balance impairment may provide further insights.

### Summary

A standardized method for determining the specific contributions of the vestibular system to age-related balance impairment has yet to be developed. Although traditional measures of vestibular function allow one to infer the integrity of the peripheral or central vestibular structures, the extent by which they capture the specific subclinical age-related changes that affect the vestibular balance pathways appears limited (Table 3). Standardized balance assessments are sensitive to acute vestibular lesions and predict fall risk, yet, fail to account for the multi-modal nature of the vestibular system. One hypothesis arising from recent basic research (17, 22, 23, 190) is that roll tilt vestibular thresholds reflect central vestibular noise that affects both perception and balance and thus may provide a method to quantify contributions of the central vestibular system to age-related balance impairment. Identifying a method to detect vestibular contributions to age-related postural instability would support efforts to screen for vestibular mediated fall risk and could potentially permit earlier implementation of targeted rehabilitative interventions.

**Table 3:** Summary of main findings.

➢The video head impulse test and measures of dynamic visual acuity correlate with imbalance in older adults; tests of the low frequency VOR (i.e., caloric irrigation and rotational chair testing) do not.
➢ Clinical and instrumented balance tests capture age-related changes in balance performance but are limited in their capacity to isolate specific vestibular contributors (canal versus otolith versus central processing, etc.) to balance dysfunction.
➢Postural responses to galvanic vestibular stimulation are attenuated in older adults yet the relevance of this finding to age-related imbalance is unclear.
➢Biased perceptions of subjective postural vertical and subjective visual vertical correlate with imbalance in older adults suggesting that age-related changes in multisensory integration may contribute to age-related imbalance.
➢Increased vestibular noise, quantified by roll tilt vestibular thresholds, is a significant predictor of sub-clinical balance impairment in asymptomatic older adults; this finding suggests that roll tilt vestibular thresholds may reflect a shared source of central vestibular rnoise affecting both the balance and perceptual pathways of older adults.

## Author Contributions

AW and DM conceptualized the manuscript. AW created the initial draft of the manuscript. OA, AC, KB, and DM contributed to the writing and editing of the manuscript. All authors contributed to the article and approved the submitted version.

## Conflict of Interest

The authors declare that the research was conducted in the absence of any commercial or financial relationships that could be construed as a potential conflict of interest.
